# Effect of Sour Cherry or Plum Juice Marinades on Quality Characteristics and Oxidative Stability of Pork Loin

**DOI:** 10.3390/foods11081088

**Published:** 2022-04-10

**Authors:** Violeta Nour

**Affiliations:** Department of Horticulture & Food Science, University of Craiova, 13 AI Cuza Street, 200585 Craiova, Romania; vionor@yahoo.com; Tel.: +40-722-791-987

**Keywords:** meat marination, sour cherry juice, plum juice, water-holding capacity, storage, lipid oxidation, color, sensory analysis

## Abstract

In this study, the potential of sour cherry and plum juices was evaluated to be used in the traditional marination of meat. Slices of pork loin were marinated for 24 h in brine of 3% NaCl or sour cherry and plum juice marinades containing 60% fruit juice while the control group consisted of non-marinated meat slices. Proximate composition, marinating loss, cooking loss, drip loss, and water-holding capacity of samples was evaluated. Changes in surface meat color (L*, a*, and b* values), pH, ammonia content, and thiobarbituric acid reactive substances (TBARS) values were monitored in pork loins during 12 days of refrigerated storage. Sensory evaluation was also conducted. There was a significant decrease in pH, moisture content, and water-holding capacity of raw meat due to fruit juice marination, resulting in marination losses and higher cooking losses compared with the control and brine-marinated samples. During cold storage, marination with sour cherry and plum juices was found to slow down the increase in TBARS values and ammonia content and the decrease in all meat sensory scores. Thus, sour cherry and plum juices may be used as marinating ingredients as they promote interesting sensory properties and improve the storage stability of pork loin.

## 1. Introduction

Marination is a traditional and industrial technique applied prior to further thermal processing that has been used in the past and is still extensively used on various types of meat and poultry products in order to improve technological and sensory meat properties [1,2]. Marination is reported to (a) increase meat yield by increasing water-holding capacity and reducing water loss during cooking, (b) enhance meat quality by increasing tenderness and juiciness, improving or preserving color, enhancing aroma and flavor, and reducing off-flavors, and (c) improve meat safety and extend the shelf life of meat by limiting the growth of bacteria [3,4]. Commercially, marination is performed by vacuum tumbling or injection but the simplest marinating method is the static soaking of the meat in a marinade solution [5,6].

Marination ingredients include salts, phosphates, organic acids, spices, antioxidants, flavor enhancers, herbs, and tenderizers [7]. The most common ingredient in marinating solutions is sodium chloride, which is known to increase the water-holding capacity of the meat due to its ability to increase the electrostatic repulsion of myofibrillar proteins, and in this way, to enlarge the space between actin and myosin filaments, allowing additional quantities of water to be retained in meat [1]. A similar effect can be achieved by the addition of phosphates, which are able to increase pH value, and food acids (lactic, citric, ascorbic, tartaric, and acetic acids) [8]. NaCl also has the ability to enhance meat flavor and juiciness as a result of the extraction of salt-soluble myofibrillar proteins and to inhibit microbial growth during storage of meat products by controlling water activity, osmotic pressure, and electrolyte balance [9]. Recently, the use of organic acids (lactic, acetic, and citric) has been reported to prevent bacterial spoilage in meat products [10,11]. Acid marination affects meat tenderness as a result of the weakening and swelling of muscle fibers and/or connective tissue and of the solubilization of collagen during cooking [12].

Following the currently increasing demand by consumers for healthy and natural food products, the recent studies focused on the use of natural ingredients and additives in the marinating process instead of synthetic ingredients [13]. Apart from vinegar, wine, and lemon juice, which are the most common natural ingredients used in acidic marinades, an increasing number of studies have tried to use fruit and vegetable juices in meat marination. Gök and Bor [14] investigated the effect of marination with various fruit and mixed vegetable juices on chemical, textural, and sensorial properties of turkey breast meat and found that marination in pomegranate and red grape juices reduced the hardness values in turkey breast meat. Unal et al. [15] investigated the effect of marinades prepared with citric acid, lemon juice, and grapefruit juice on the sensory, textural, and microstructure characteristics of poultry meat. Demir et al. [16] reported that marination in onion juice reduced the lipid oxidation in beef and increased the general liking score in the sensorial evaluation while Kadıoğlu et al. [17] found that marination with pineapple fruit juice improved the tenderness of spent hen meats. Dairy products such as kefir, yoghurt, buttermilk, and acid whey have been also tested for marinating purposes on pork loin, turkey, and pheasant meat [4,6,18].

Sour cherries (*Prunus cerasus* L.) are popular fruits in professional and household fruit growing due to the bright cherry flavor, refreshing acidity, and vivid dark-red color [19]. They are also known as a valuable natural source of some bioactive compounds with high antioxidant activity and beneficial influence on human health [20]. However, the high acidity and astringency of sour cherries limits their fresh consumption [21] and as a result, they are usually processed in different sugar-preserved products such as syrups, jams, jellies, marmalades, etc. Sour cherries have a high content of organic acids (mainly L-malic acid), minerals (potassium, magnesium, copper, and manganese), and polyphenolic compounds, especially anthocyanins, which are the major contributors to their antioxidant activity [22]. Apart from anthocyanins, sour cherries also contain other important phenolic compounds exerting strong antioxidant activity [23,24].

Plums (*Prunus domestica* L.) are popular fruits cultivated all over the world that have received special consideration in recent years due to their antioxidant activity and health-promoting properties [25]. Plums have been described as one of the richest source of phenolic compounds [26] which are known to possess various pharmacological effects including antioxidant, antimicrobial, anti-inflammatory, antimutagenic, antidiabetic, and neuroprotective properties [27,28]. Phenolic compounds are considered to contribute to the sensory qualities of the fruits such as color, sour taste, characteristic astringency, and distinctive fruity flavor. The anthocyanins are located mostly in the fruit skin and they are responsible for the purple color of plums [29]. Moreover, plums provide a source of important minerals including potassium, calcium, magnesium, and boron [30]. 

To address the increased demand for the use of new natural ingredients in meat processing, the present study was conducted to investigate the impact of 24 h marinating in sour cherry juice and plum juice based marinades on physicochemical and technological meat characteristics, lipid oxidation degree, and sensory properties of pork loin during 12 days of refrigerated storage.

## 2. Materials and Methods

### 2.1. Materials

The *Longissimus dorsi* muscle of Pietrain pig carcasses at 24 h postmortem were obtained from a local abattoir the same day of the experiment, then transported to the laboratory under refrigerated conditions (4 °C). All trimmable fat and connective tissue were removed. Muscles were cut into 2 cm thick slices weighing about 100 g, perpendicular to the direction of the muscle fibers. The sour cherries and the plums to be used for the preparation of marinades were procured from a local market. Mature and undamaged fruits were selected and stored at 4 °C. 

### 2.2. Preparation of Fruit Juices and Marinades

On the day of the experiment, the fruits were manually stoned and pressed to liberate the juice using a home-type juice extractor (Tefal Easy Fruit ZE610D38, China, 800W). The extraction juices were filtered through two thicknesses of cheesecloth and the resulting juices were used for the preparation of marinades. Sour cherry and plum juices were produced daily prior to the marination process. Two different marinades were prepared by mixing fruit juice (sour cherry and plum juice, respectively), water, and salt (sodium chloride) in the ratio 60:37:3 (*w*/*w*/*w*). Separately, a brine of 3% (by wt) NaCl was prepared.

### 2.3. Marinating Procedure

Before use, the marinades were cooled to the temperature of 8 °C ± 1 °C. Brine and marinades were added to the meat slices: 3% salt solution (S), sour cherry marinade (SCM), and plum juice marinade (PM). The control group (C) consisted of meat slices not subjected to the marinating process (without any addition). The prepared meat slices (*n* = 80) were randomly assigned to C, S, SCM, and PM groups (*n* = 20 in each group), weighed and placed in separate disposable plastic containers (500 mL capacity) covered by lid, each containing one slice. The salt solution and the marinades were applied at a 1:1 ratio of pork loin weight to liquid weight, and the pork loins were marinated for 24 h at 4 °C. Before and after the marinating process, the samples were individually weighed with an accuracy of 0.1 g (Radwag WLC 6/A2, Radom, Poland). After treatment, the samples were stored at 4 °C for 12 days. TBARS values, surface CIELab color parameters, pH, and ammonia content were evaluated at 0, 3, 6, 9, and 12 days of post-treatment storage. 

### 2.4. Marinating Loss

Meat samples were removed from marination containers, slightly wiped with paper towels to remove excess surface moisture, and reweighed. Marination loss was calculated as follows: % marinating loss = (weight of raw sample—weight of marinated sample)/weight of raw sample × 100.

### 2.5. Drip Loss

The drip loss was determined as described by Honikel [31]. Non-marinated and marinated pork loins were weighed and placed into a sealed polyethylene bag. After storage for 24 h at 5 °C, the samples were reweighed. Drip loss was calculated as follows: % drip loss = [(initial weight—final weight)/initial weight] ×100.

### 2.6. Cooking Loss

Non-marinated and marinated pork loins were weighed (accuracy = 0.1 g) and processed in an electric oven (Beko, Istanbul, Turkey) at 180 °C until 75 ± 1 °C was reached in the center. The temperature was measured with a digital thermometer ((TP 3001, Qingdao, Shandong Province, China). After cooling, the samples were reweighed and cooking loss was calculated as follows: % cooking loss = [(raw weight—cooked weight)/raw weight] ×100.

### 2.7. Proximate Composition

The meat raw materials were analyzed for moisture, fat, and protein content according to the methods of the AOAC (1990). Moisture content was determined based on moisture loss at 105 °C in a drying oven (Memmert ULM500, Uden, The Netherlands), the fat content was determined by the Soxhlet method using a Soxhlet automatic extraction system (SER 148/3, Velp Scientific, Usmate, Italy), and the protein content was determined by the Kjeldahl method using an automated nitrogen analyzer (UDK 149 Velp Scientific, Milan, Italy).

### 2.8. Water-Holding Capacity

The water-holding capacity (WHC) of meat samples was determined based on the technique of Hamm [32] as described by Rupasinghe et al. [7]. Pieces of meat samples (2 g) were placed between two sheets of Whatman No. 1 filter paper on acrylic plates. A 10 kg weight was placed on the top plate and left for 5 min. Each sample was weighted after compression and WHC was calculated using the following equation:

WHC (%) = 100 − [(initial weight of meat samples − final weight of meat samples) × 100/initial weight of meat samples].

### 2.9. Color Measurement

Color changes during storage were monitored by measuring the lightness (L*), redness (a*), and yellowness (b*) values of the CIELab system at 0, 3, 6, 9, and 12 days of post-treatment storage using a PCE-CSM1 colorimeter calibrated against a white standard. Color was measured on the surface area of non-marinated and marinated pork loins (three samples from each treatment with six random readings on each sample). In addition, color parameters were determined on the surface of pork loin samples after marination for 24 h and cooking.

### 2.10. pH Measurement

Meat samples (5 g) were grinded by a blender (Braun MQ5137BK, 750W). The minced samples were homogenized with 50 mL of distilled water for 3 min. The pH of fruit juices, marinades, and meat homogenates was determined using a pH meter (Hanna HI255, Padova, Italy) after calibration with pH 4 and pH 7 buffers.

### 2.11. Total Phenolic Content

The total phenolic content of the fruit juices and of the marinades was assessed using the Folin Ciocalteu method as described by Singleton et al. [33]. Fruit juices and marinades (3 mL) were extracted with 10 mL methanol in a Bandelin Sonorex Digital 10P ultrasonic bath (Bandelin Electronic GmbH, Berlin, Germany) for 60 min at ambient temperature. After extraction, the samples were centrifuged for 5 min at 4200 rpm. Supernatants were collected, filtered, and used for the assays. Aliquots of extracts (0.1 mL) were mixed with 5 mL of distilled water and 0.5 mL of Folin Ciocalteu reagent. After 30 s to 8 min, 1.5 mL of sodium carbonate (20 % *w*/*v*) was added and the final mixture was made up to a volume of 10 mL with distilled water. The same procedure was also applied to the standard solutions of gallic acid. The absorbance was measured at 765 nm on a Varian Cary 50 UV spectrophotometer (Varian Co., Palo Alto, CA, USA) after incubation for 30 min at 40 °C. Results were expressed as mg of gallic acid equivalents (GAE) per 100 mL.

### 2.12. DPPH Radical Scavenging Activity

Antioxidant activity of the fruit juices and marinades was measured using a DPPH (2,2-diphenyl-1-picrylhydrazyl) procedure described by Hatano et al. [34]. Briefly, aliquots of extracts (50 μL) were mixed with 3 mL of a 0.004% (*v*/*v*) DPPH methanolic solution. After incubation in darkness for 30 min, the absorbance was read at 517 nm using a Varian Cary 50 UV spectrophotometer (Varian Co., Palo Alto, CA, USA). The ability of the sample to scavenge the DPPH radical was calculated as follows: DPPH scavenging activity (%) = [1 − (absorbance of sample/absorbance of control)] × 100. Trolox (6-hydroxy-2,5,7,8-tetramethylchroman-2-carboxylic acid) was used as a standard reference and results were expressed as mmol Trolox equivalents (TE) per 100 mL.

### 2.13. Thiobarbituric Acid Reactive Substances (TBARS) Value

TBARS of pork loins were determined using the spectrophotometric method described by Witte et al. [35] with minor changes. Briefly, 5 g homogenized sample was vortexed for 2 min with 12.5 mL of 20% trichloroacetic acid then diluted to 25 mL with cold distilled water. After centrifugation for 5 min at 1792× *g*, 5 mL of extract was mixed with 5 mL of 0.02 M 2-thiobarbituric acid and warmed in a boiling water bath for 35 min. After cooling in refrigerated water, the absorbance was recorded at 532 nm in a Varian Cary 50 UV spectrophotometer (Varian Co., Palo Alto, CA, USA). A calibration curve was constructed using 1,1,3,3-tetraethoxypropane (Sigma-Aldrich) standard solutions. TBARS values were expressed as mg of malondialdehyde (MDA) per 100 g of meat sample.

### 2.14. Ammonia Content

Ammonia (easily hydrolysable nitrogen) content of pork loins was assessed by the distillation method as presented by Parris and Foglia [36] with slight modifications. Sample (10 g) was placed in a 500 mL round bottom flask containing 200 mL of distilled water, 3 g of MgO as catalyzer, and a few drops of anti-foam. The mixture was distilled and the distillate was collected in 50 mL of 4% boric acid before titration with 0.1 N HCl using Tashiro as an indicator. Ammonia content was expressed as mg NH_3_ per 100 g sample. The results were interpreted as follows: fresh meat = 6–14 mg/100 g; meat at risk of decomposition = 14–20 mg/100 g; meat affected with initial degradation process = 20–42 mg/100 g; markedly tainted meat (rotting) = > 42 mg/100 g.

### 2.15. Sensory Evaluation

Sensory attributes of the non-marinated and marinated pork loins were evaluated initially as well as after 3, 6, 9, and 12 days of refrigerated storage. The pork loins were cooked as described above, then cut and served randomly to panelists. The cooked samples were evaluated on a 9-point scale for color acceptability (1 = very unacceptable; 9 = very acceptable), tenderness (1 = very tough; 9 = very tender), juiciness (1 = very dry; 9 = very juicy), taste (1 = very unacceptable; 9 = very acceptable), flavor intensity (1 = very weak; 9 = very strong), and overall acceptability (1 = very unacceptable; 9 = very acceptable). Water and bread were served between samples as palate cleansers. The panel consisted of twelve trained members from the University of Craiova food science staff and post graduate students.

### 2.16. Statistical Analysis

The entire experiment was repeated three times (four pork loins per each batch). Three replicates were performed for each analysis and data were reported as means ± standard deviation. Data were subjected to analysis of variance (ANOVA) and means were compared by the least significant difference (LSD) test (*p <* 0.05). Statistical analyses were carried out using Statgraphics Centurion XVI software (StatPoint Technologies, VA, USA).

## 3. Results

### 3.1. Soluble Solid Content, pH, Titratable Acidity, Total Phenolic Content, and Antioxidant Activity of Fruit Juices and Marinades

Soluble solid content, pH, titratable acidity, total phenolic content, and DPPH antioxidant activity of fruit juices and marinades are presented in Table 1. Sour cherry juice presented a significantly higher (*p* < 0.05) total phenolic content and DPPH antioxidant activity compared with plum juice, while the latter presented the highest soluble solid content. The results on total phenolic content and antioxidant activity of sour cherry juice were in line with the data reported by Khoo et al. [37] in thirty-four sour cherry cultivars. Other previous studies have shown that sour cherries are potentially good raw material for functional foods because of considerable content of polyphenols, especially anthocyanins responsible for their dark red color, distributed in both fruit skin and flesh [20,22]. In their turn, plums are often touted as functional foods due to their high levels of phenolics and other antioxidant compounds [30]. The major phenolic compounds in plums are hydroxycinnamic acids such as chlorogenic, neochlorogenic, p-coumaric, caffeic, and ferulic acid, flavonoids including rutin (quercetin 3-rutinoside) and catechin, and anthocyanins such as cyanidin 3-rutinoside, cyanidin-3-glucoside, and peonidin 3-rutinoside [38,39]. The results found for plum juice in the present study (129.34 mg GAE/100 g and 0.54 mmol Trolox/100 g for total phenolic content and antioxidant activity, respectively) are in good agreement with previous publications. Rupasinghe et al. [40] reported total phenolic contents ranging from 86 to 413 mg GAE/100 g among twenty European plum genotypes while Slimestad et al. [41] found antioxidant activity between 0.290 and 0.814 mmol Trolox/100 g fresh weight in six plum cultivars. The lowest pH value was found in the sour cherry juice (3.06). pH-values between 3.07 and 3.30 have been previously reported in juices from different sour cherry cultivars [19].

The results for marinades were closely correlated with those obtained for juices. As fruit juices have contributed only 60% to marinades, total phenolic content and antioxidant activity of marinades were lower than the juices from which they were made. The sour cherry marinade presented a higher total phenolic content and antioxidant activity and a lower soluble solid content as compared to the plum marinade. As well, the pH values of the marinade solutions were higher than that of the juices used to prepare them. The lowest pH value was measured in the sour cherry marinade (3.28 as against 3.54 in the plum marinade).

### 3.2. Proximate Composition, Marinating Loss and Cooking Loss

The proximate composition and pH of pork loin used in this study were as follows: moisture content, 74.69 ± 0.65%; protein, 21.58 ± 0.27%; fat, 3.75 ± 0.37%; and pH, 5.54 ± 0.03. The proximate composition of the control samples and pork loin samples after marination for 24 h at 4 °C is shown in Table 2. 

Marinating the meat samples for 24 h in 3% NaCl solution resulted in a weight gain of 5.18% while marination in fruit juice based marinades determined a marination loss of 2.13% and 2.61% for sour cherry marinade and plum marinade, respectively. As a result, the moisture content of meat samples marinated with fruit juices was significantly lower (*p* < 0.05) compared with the samples soaked in 3% sodium chloride solution. Similar decreases of the moisture content was reported by Gök and Bor [14] in turkey breast meat after marination for 24 h in fruit juices. The marination losses were probably due to the osmotic dehydration of the meat consisting in the partial diffusion of the water from the meat tissue with lower sugar concentration into the surrounding fruit juice marinade with higher sugar concentration as a result of the osmotic pressure exerted by the marinade [42]. Some previous studies reported a weight gain of the meat after soaking in sour marinades, correlated with the increase of the meat’s water-holding capacity. These properties have been attributed to the swelling and enhanced extractiveness of myofibrillar proteins determined by the increase in ionic strength and the decrease in pH [43,44]. However, other studies have also recorded marinating losses in chicken wings marinated with fruit juices for 24 h [7] or in pork loin marinated in fermented dairy products for 48 h [4].

### 3.3. Evolution of pH Values

**Figure 1:**
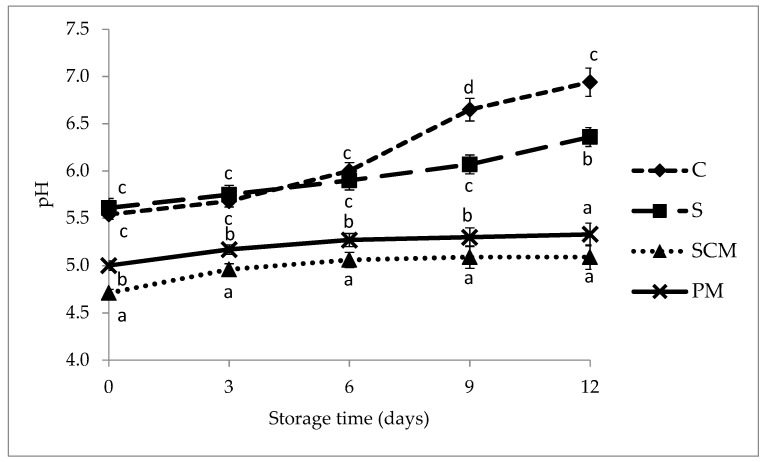
Evolution of pH values of control and marinated pork loins during 12 days of refrigerated storage. C, Control (without marination); S, samples marinated in 3% NaCl solution; SCM, samples marinated in sour cherry juice based marinade; PM, samples marinated in plum juice based marinade; ^a–d^ Values with different superscript letters for the same storage time differ significantly (*p* < 0.05).

Evolution of pH values after marination of the meat samples during 12 days of refrigerated storage is shown in Figure 1. Marinating in fruit-juice-based marinades significantly decreased pH values of the meat (*p* < 0.05) as a result of the low pH of the marinades used (Table 1). Other previous studies reported also that the acidity of the marinated product was strongly influenced by the acidity of the marinade [6,14,17]. 

Right after the marinating process, the lowest pH was registered in sour-cherry-juice-marinated pork loins (4.71) followed by the plum-juice-marinated samples (5.02) and the control samples (5.54), while the samples marinated in 3% NaCl solution registered the highest pH (5.61).

pH value is an important parameter affecting the physico-chemical, biochemical, technological, and microbiological characteristics of the meat and their evolution during storage. pH values increased slightly during the entire storage period in all meat samples due to the accumulation of alkaline nitrogenous compounds such as amines and ammonia, as a result of enzymatic reactions and microbial attack [45]. In the first six days of storage, values in samples soaked for 24 h in 3% NaCl solution were close to the values of control (non-marinated) samples, while in the next six days, the pH of the control samples was significantly higher than the brine-marinated samples. Pork loin marinated with sour cherry juice showed the lowest pH values throughout the storage period (*p* < 0.05), followed by the samples marinated with plum juice. At the end of the storage period, the highest pH values were determined in control pork loins.

### 3.4. Water-Holding Capacity, Drip Loss, and Cooking Loss

Table 3 shows the water-holding capacity, drip loss, and cooking loss of pork loin samples after the marination treatment. The water-holding capacity is one of the most important technological characteristics that influence the quality and yield of processed meat and meat products. As expected, marinating in 3% sodium chloride solution alone determined a significant increase in the water-holding capacity of the meat samples. This effect is attributed to the promotion of a stronger dissociation of the acidic groups than the amine groups by sodium chloride, which consequently moves the isoelectric point of the proteins toward lower pH values [18]. The addition of 60% fruit juice in the marinade determined a slight decrease of this parameter. The meat pH value is a determining factor of the water-holding capacity [46]. The lower water-holding capacity in samples marinated in fruit juice marinades may be attributed to the strong effect of their low pH values (4.71 and 5.00 for SCM and PM meat samples, respectively) which were very close to the isoelectric point of the meat proteins (pH 5.0–5.2), resulting in the decrease in the net charge and of the electrostatic repulsion between proteins and hence of the number of water molecules combining with them. Some previous studies have also reported that bringing the pH to values near the isoelectric point of the meat proteins determined a significant decrease of the meat water-holding capacity [12,47]. Rupasinghe et al. [7] found also a lower water-holding capacity in chicken wings marinated with mango juice for 24 h compared with the control unmarinated chicken wings.

Cooking loss decreased in brine-marinated samples and slightly increased in fruit-juice-marinated samples compared with control samples; however, the differences were not significant (*p* < 0.05). Kadıoğlu et al. [17] and Serdaroğlu et al. [47] reported also higher cooking loss values in marinated chicken meat samples than in untreated samples.

The highest drip losses were found in PM and SCM samples while the lowest was found in S samples. As previously found in other studies, a significant decrease in the drip loss was observed in pork loin marinated with sodium chloride solution [48], while fresh meat with lower pH was associated with higher drip losses [49]. As previously stated, drip loss and cooking loss were directly influenced by the water-holding capacity of meat [48].

### 3.5. Evolution of Ammonia Content

**Figure 2:**
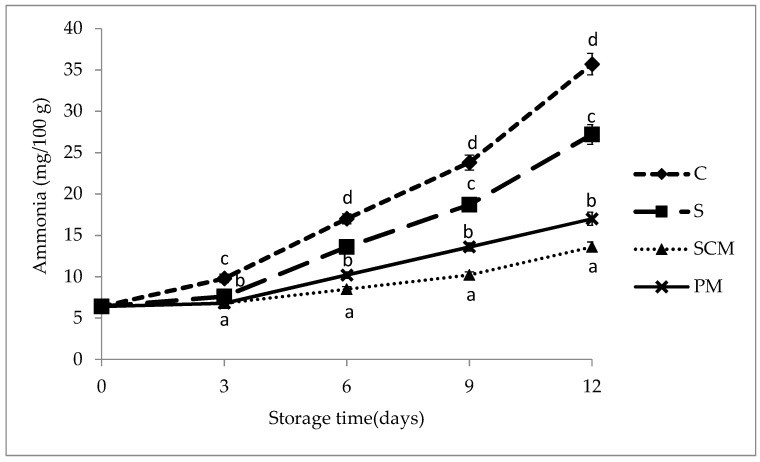
Evolution of ammonia (easily hydrolysable nitrogen) content (mg/100 g) in control and marinated pork loins during 12 days of refrigerated storage. ^a–d^ Values with different superscript letters for the same storage time differ significantly (*p* < 0.05).

Figure 2 presents the evolution of ammonia (easily hydrolysable nitrogen) content in control and marinated pork loins during storage. The ammonia content significantly increased during storage in all meat samples as a result of the deamination of amino acids after degradation of protein by the enzymatic and spoilage microbial action [50]. Generally, the meat ammonia content is correlated with the degree of contamination with microorganisms and their biochemical activity as well as with the sensory changes of the meat [51]. High ammonia values were recorded in control (non-marinated) samples already after nine days of storage, suggesting the beginning of the degradation process and the potential risk of meat spoilage. After 12 days of storage, the ammonia content amounted to 35.7 mg/100 g in control (non-marinated) samples, 27.2 mg/100 g in S samples, and 13.6 mg/100 g and 17 mg/100 g in SCM and PM samples, respectively. The ammonia content in fruit-juice-marinated samples was lower throughout the storage period compared to the control and brine-marinated samples, probably due to slowing down of the microorganisms multiplication processes in an environment with more acidic pH. Some previous studies demonstrated that organic acid and fruit juice marination increased the microbiological safety of the meat products [18,52].

### 3.6. Lipid Oxidation

**Figure 3:**
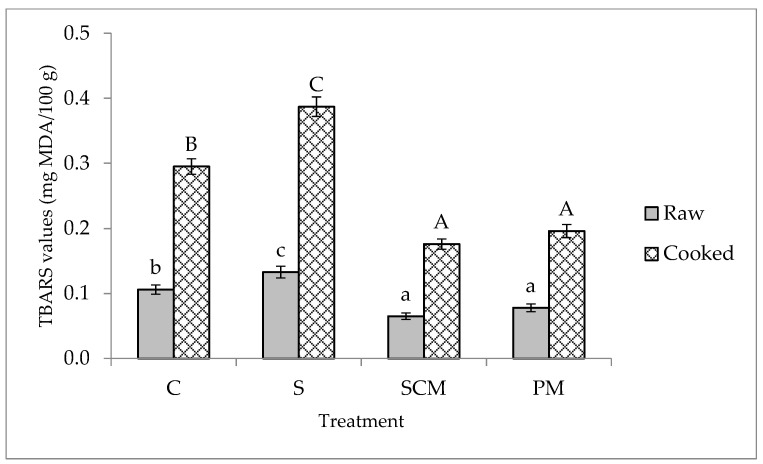
TBARS values of raw and cooked control and marinated pork loins after 12 days. refrigerated storage. Different lowercase letters indicate significant differences between raw samples (*p* < 0.05) while different uppercase letters are indicative of significant differences between cooked samples (*p* < 0.05).

Lipid oxidation is a major cause of quality deterioration in meat products during storage that lowers their nutritional and functional properties and negatively affects their sensory characteristics. Lipid oxidation determines off-flavors, color, and texture defects and reduces the shelf-life of meat products due to the formation of toxic compounds including reactive oxygen species, free radicals, and hydroperoxides. TBARS values reflect the content of secondary products of lipid oxidation, mainly aldehydes and carbonyls, which may contribute to the development of off-flavors in oxidized meat and meat products [2,53]. Therefore, in the current study, TBARS values were measured after 12 days of refrigerated storage in the uncooked and cooked pork loin samples (Figure 3). 

The TBARS values increased in all treatments over time, both in raw and cooked pork loins. Marinating in 3% NaCl solution resulted in higher TBARS values compared to the controls, in agreement with previous studies demonstrating that salt addition increases the intensity of oxidative reactions [54,55]. In addition, TBARS values were significantly higher in cooked samples as compared with the raw samples, probably because cooking induced an additional oxidation, as previously demonstrated [56]. After 12 days of storage and cooking, TBARS values were close to 0.3 mg MDA per 100 g in control samples and exceeded this threshold in brine-marinated samples. However, the TBARS values in fruit-juice-marinaded samples were significantly lower than in control samples (*p* < 0.05). Raw samples marinated in fruit juices were well preserved in terms of oxidative changes as TBARS values did not exceed 0.1 mg of malondialdehyde (MDA) per 100 g after 12 days of refrigerated storage (Figure 3). This may be due to the effectiveness of phenolic compounds from fruit juices to prevent or decelerate lipid oxidation associated with their free radical-scavenging activity. Similar effects were reported by Demir et al. [16] in beef marinated with onion juice or by Rupasinghe et al. [7] in vacuum-packed chicken wings marinated with mango, pineapple, and plum juices. The lowest TBARS values were recorded in pork loins marinated in sour cherry based marinade, a finding which is supported by the highest antioxidant activity and total phenolic contents found in sour cherry juice in this study.

### 3.7. Color

**Figure 4:**
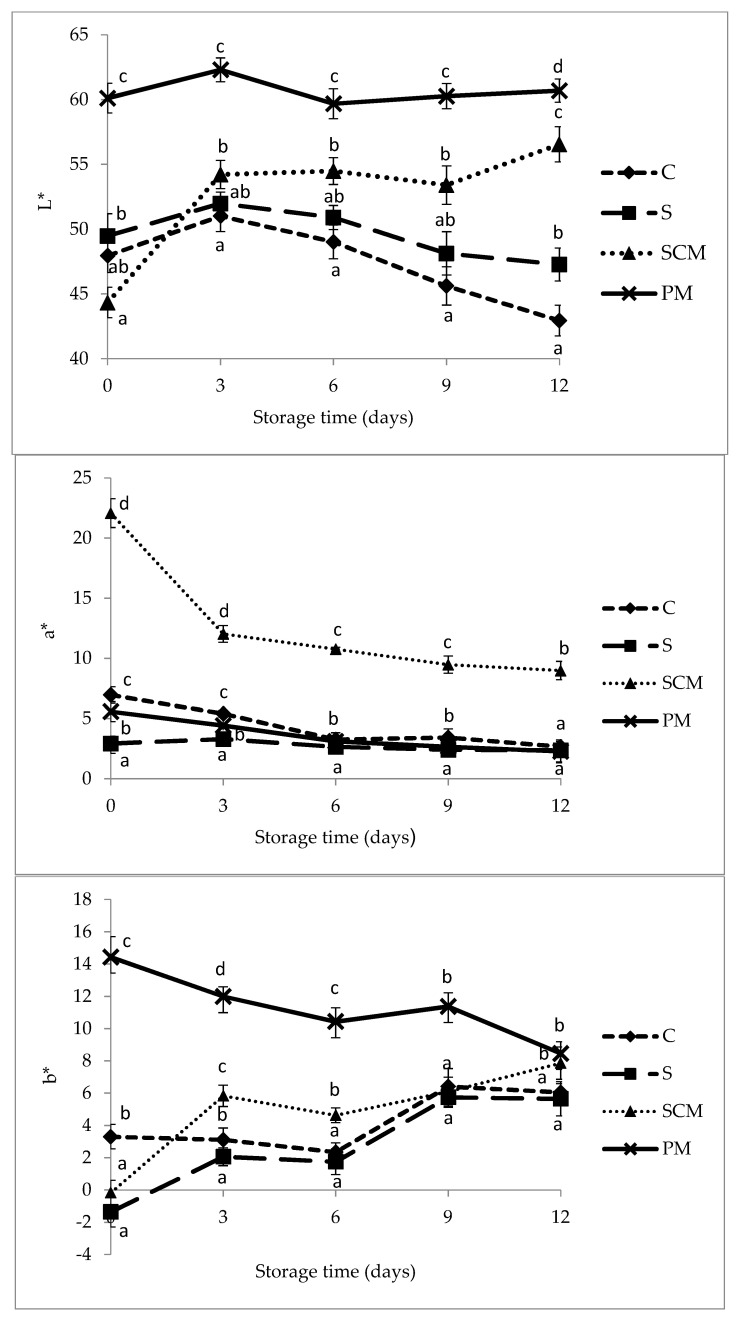
Changes in the surface meat color (L*, a*, and b* values) of control and marinated pork loins during 12 days of refrigerated storage. ^a–d^ Values with different superscript letters for the same storage time differ significantly (*p* < 0.05).

Color is an important sensory attribute in the appeal of meat and meat products to consumers. Results of our study showed that immersing in fruit juice marinades significantly modified the color of pork loin samples and its evolution during storage (Figure 3). Marinating in plum juice contributed to the significant color lightening (increase of the L* parameter) of raw marinated pork loins compared to control (non-marinated) samples (Figure 4). 

Further, the L* parameter of these samples did not register significant fluctuations during 12 days of storage. Similarly, some previous studies reported that the use of lemon, pineapple, and grapefruit juice marinades contributed to the color lightening of raw and roasted meat marinated products compared with the non-marinated samples [17,18,47]. The color lightening of the acidic marinated products has been attributed to the presence of the extracellular water during marinating and to the swelling of muscle proteins at low pH and ionic strength [18].

Marinating in sour cherry juice determined the decrease of the L* value compared to control (non-marinated) samples (Figure 3; Table 4) due to the coloration provided by the anthocyanin pigments from the sour cherry juice. A similar decrease in the lightness parameter L* was noted by Gök and Bor [14] after 24 h of marination in some fruits and mixed vegetable juices. Afterwards, L* increased during storage probably due to the decrease in the anthocyanins content at the surface as a result of the permeation of the anthocyanin compounds from the surface into the meat and to the blanching determined by the degradation of the anthocyanins from the surface. 

In control and brine-marinated samples, lightness (L* values) recorded a slight increase in the first days of storage and after that it decreased steadily. However, at the end of the storage period, lightness was higher in brine-marinated samples compared to control samples.

The a* value (redness) was strongly influenced by the color coming from the marinating fruit. Marination in sour cherry juice caused an outstanding increase of the a* values as expected, considering the high anthocyanins content of sour cherry juice and their red color in acidic conditions. Similarly, Gök and Bor [14] reported an increase in a* values after turkey breast meat was marinated in black mulberry, black carrot, and pomegranate juices, which are rich in natural red pigments. Plum juice and brine marination resulted in a slight decrease in the a* values compared to the control sample. Slight decreases in the a* values were previously reported after 24 h red grape juice or grapefruit juice marination of turkey breast meat [14,47] compared to the control sample. Redness (a*) of sour-cherry-juice-marinated samples decreased sharply in the first three days of storage and continued to decline slightly over the rest of the storage period as a result of the superficial degradation of anthocyanins and their permeation into the meat. In the other samples, a* values registered slight decreases during the storage period.

Marinating pork loin in plum juice based marinade resulted in a high increase in the surface b* values compared with the control samples, as previously found in meat samples marinated in red grape and black mulberry juices [14] or pineapple juice [17]. However, in brine and sour-cherry-juice-marinated samples, b* values decreased as a result of marinating but increased continuously during the 12 days of storage.

The changes in meat color as a result of the heat treatment are presented in Table 4. After cooking, the L* and b* values of all (marinated and control) samples increased. Similar effects of cooking on L* values of fruit-juice-marinated meat samples were reported in previous studies [14,47]. Higher b* values of cooked meat samples compared to raw meat may be due to the formation of metmyoglobin, a brown pigment, in the final product.

The a* values significantly decreased after cooking in sour-cherry-marinated samples, as previously reported by Gök and Bor [14] in turkey breast meat samples marinated in black carrot juice. Heat treatment of meat determined degradation of myoglobin and as a result, decreases of the a* values. In addition, heat treatment caused the degradation of anthocyanins in these samples, which further contributed to the decrease in the a* values. In plum-juice-marinated samples, a* values increased slightly after marinating, as previously found in turkey meat marinated in black mulberry, red grape, or pomegranate juices [14]. The antioxidant compounds from plum juice can limit the oxidation of myoglobin, thus minimizing the changes in a* values [4].

### 3.8. Sensory Evaluation

The results of the sensory evaluation of pork loin samples during 12 days of refrigerated storage are shown in Table 5. 

In terms of color, pork loins marinated with sour cherry juice had the lowest acceptance score compared with the other treatments (*p* < 0.05) because of the dark-red color of the meat surface, which was hard to accept by some panelists, while others found it appealing. The scores for color did not differ significantly (*p* < 0.05) between control and brine-treated samples while the yellower color of plum-juice-marinated samples was less appreciated than control samples. As expected, scores of all sensory attributes including those for color decreased during storage. However, the decrease in the color score in time was slower in the samples marinated in fruit juices than in control and brine-immersed samples. As a result, after 12 days of storage, the color score of fruit-juice-marinated samples was significantly higher (*p* < 0.05) than the color score of the control samples.

Marination affected tenderness and juiciness of pork loins, as appreciated by the panelists. After treatment, tenderness score was highest in brine-treated pork loins followed by control, sour-cherry-juice-, and plum-juice-marinated samples (Table 5). Previously, the subjective tenderness scores of sensory panelists were negatively correlated with the hardness of texture profile analysis [57]. Some studies reported lower hardness values in turkey meat marinated in grapefruit juice [47] or lemon juice [18]. Gök and Bor [14] found that marinating in black mulberry, red grape, and pomegranate juice decreased the hardness of the turkey breast meat while marinating in mixed vegetable juice and black carrot juice resulted in higher (*p* < 0.05) hardness values. 

Generally, lowering the pH in meat can reduce the hardness of muscle tissue by weakening electrostatic interactions between myofibrillar protein chains and the connective tissue by softening the stromal proteins. However, as previously presented, the pH values (4.71 and 5.00 for SCM and PM meat samples, respectively) were close to the isoelectric point of the meat proteins (pH 5.0–5.2), resulting in the decrease in the net charge and electrostatic repulsion between proteins. On the other hand, in the acidic environment created by marinating in fruit juices, the activity of cathepsins is raised, as their optimal pH is in the range from 3.5 to 5.0.

The mean juiciness scores were significantly higher for brine-marinated samples than control samples. This was expected as NaCl provide better water absorption and holding capacity to the meat. Fruit-juice-marinated samples registered lower juiciness scores than brine-marinated samples, in good agreement with their lower values of the water-holding capacity and their higher cooking losses (Table 3).

In terms of taste, the score was significantly lower in control samples as a result of the lack of the salty taste. The mean taste score was lower in sour-cherry-marinated samples compared with the brine-marinated samples due to their slightly sour taste while the highest taste score was given to the samples marinated in plum juice probably due to the sweeter taste coming from the higher sugar content of the plum juice. The fruity flavor of juice-marinated samples was also appreciated by panelists. At the end of the storage period, the scores for taste and flavor were significantly higher in fruit-juice-marinated samples, probably due to the lower intensity of both microbiological and oxidative degradation processes in these samples compared to the control and brine-marinated samples.

After treatment, the results on general acceptability were favorable to fruit juice marination, particularly when using plum juice as a marinade ingredient. By the end of the storage period, samples marinated in fruit juices showed significantly higher scores of acceptability compared to the control, which probably resulted from the ability of fruit juice marinades to reduce lipid peroxidation and microbial spoilage of the meat.

## 4. Conclusions

The use of sour cherry and plum juice in marinades at a 60% level had a significant (*p* < 0.05) effect on reducing the pH, moisture content, and water-holding capacity of raw marinated meat, resulting in marination losses of 2.13% and 2.61 % for sour cherry marinade and plum marinade, respectively. Cooking losses were also slightly higher in fruit-juice-marinated samples compared with control samples. However, using sour cherry and plum juices as marinade agents limited the increase in the ammonia content and slowed down the decrease in the sensorial scores during storage compared with control and brine-marinated samples. Moreover, marinating in sour cherry and plum juices improved the lipid oxidative stability of pork loins due to the high natural antioxidant activity and total phenolic content found in these juices. The use of sour cherry and plum juices to marinate meat can be an appealing alternative to the commonly used marinades.

## Figures and Tables

**Table 1:** Soluble solid content (%), pH, titratable acidity (g malic acid/100 mL), total phenolic content (mg GAE/100 mL), and DPPH antioxidant activity (mmol Trolox/100 mL) of fruit juices and marinades ^∗^.

Samples	Soluble Solid Content	pH	Titratable Acidity	Total Phenolic Content	DPPH Antioxidant Activity
Sour cherry juice	15.14 ± 0.34 ^a^	3.06 ± 0.02 ^a^	1.36 ± 0.16 ^b^	350.45 ± 12.23 ^b^	1.50 ± 0.09 ^b^
Plum juice	16.54 ± 0.28 ^b^	3.38 ± 0.03 ^b^	0.97 ± 0.08 ^a^	129.34 ± 7.09 ^a^	0.54 ± 0.02 ^a^
Sour cherry marinade	12.72 ± 0.24 ^a^	3.28 ± 0.03 ^a^	0.82 ± 0.05 ^b^	203.41 ± 9.77 ^b^	0.87 ± 0.04 ^b^
Plum marinade	13.58 ± 0.18 ^b^	3.54 ± 0.02 ^b^	0.60 ± 0.04 ^a^	78.76 ± 4.11 ^a^	0.34 ± 0.02 ^a^

^∗^ Values with different superscript letters for juices in the same column differ significantly (*p* < 0.05); Values with different superscript letters for marinades in the same column differ significantly (*p* < 0.05).

**Table 2:** Proximate composition, marinating loss, and cooking loss of control and marinated pork loin samples ^∗^.

Traits (%)	C	S	SCM	PM
Moisture	72.22 ± 0.54 ^a^	74.56 ± 0.93 ^b^	71.58 ± 0.65 ^a^	71.60 ± 0.48 ^a^
Protein	21.86 ± 0.48 ^b^	20.93 ± 0.36 ^a^	21.28 ± 0.27 ^ab^	21.44 ± 0.45 ^ab^
Fat	3.96 ± 0.24 ^a^	3.83 ± 0.31 ^a^	4.08 ± 0.18 ^a^	4.26 ± 0.28 ^a^
Marinating loss	2.91 ± 0.35 ^b^	−5.18 ± 0.54 ^a^	2.13 ± 0.34 ^b^	2.51 ± 0.41 ^b^
Cooking loss	33.72± 1.66 ^a^	32.68 ± 0.69 ^a^	32.34 ± 1.46 ^a^	34.31 ± 0.96 ^a^

^∗^ C, Control (without marination); S, samples marinated in 3% NaCl solution; SCM, samples marinated in sour cherry juice based marinade; PM, samples marinated in plum juice based marinade; ^a,b^ Values with different superscript letters in the same raw differ significantly (*p* < 0.05).

**Table 3:** Water-holding capacity, drip loss, and cooking loss of control and marinated pork loin samples ^∗^.

Traits (%)	C	S	SCM	PM
Water-holding capacity	75.89 ± 1.95 ^ab^	77.60 ± 1.34 ^c^	74.57 ± 1.63 ^a^	75.29 ± 0.93 ^ab^
Drip loss	5.75 ± 0.22 ^b^	3.23 ± 0.18 ^a^	5.97 ± 0.34 ^b^	6.10 ± 0.36 ^b^
Cooking loss	33.72 ± 1.66 ^a^	32.68 ± 0.69 ^a^	34.34 ± 1.46 ^a^	34.81 ± 0.96 ^a^

^∗^ C, Control (without marination); S, samples marinated in 3% NaCl solution; SCM, samples marinated in sour cherry juice based marinade; PM, samples marinated in plum juice based marinade; ^a–c^ Values with different superscript letters in the same raw differ significantly (*p* < 0.05).

**Table 4:** Color parameters of raw and cooked pork loin samples ^∗^.

Traits	Parameter	C	S	SCM	PM
Raw	L *	47.95 ± 1.30 ^abA^	49.48 ± 1.71 ^bA^	44.34 ± 1.18 ^aA^	60.11 ± 1.15 ^cA^
	a *	6.95 ± 0.68 ^cB^	2.93 ± 0.62 ^aA^	22.08 ± 1.20 ^dB^	5.58 ± 0.82 ^bA^
	b *	3.30 ± 0.76 ^bA^	−1.36 ± 0.94 ^aA^	−0.17 ± 0.77 ^aA^	14.44 ± 1.26 ^cA^
Cooked	L *	69.95 ± 3.26 ^cB^	63.53 ± 2.87 ^bB^	55.00 ± 2.42 ^aB^	62.31 ± 1.65 ^bA^
	a *	5.22 ± 0.67 ^aA^	6.29 ± 0.49 ^bB^	10.06 ± 0.51 ^dA^	6.91 ± 0.24 ^cB^
	b *	9.67 ± 0.55 ^bB^	8.48 ± 0.50 ^aB^	9.33 ± 0.44 ^bB^	16.56 ± 0.53 ^cB^

^∗^ C, Control (without marination); S, samples marinated in 3% NaCl solution; SCM, samples marinated in sour cherry juice based marinade; and PM, samples marinated in plum juice based marinade. Different lowercase letters indicate significant differences between treatments (*p* < 0.05) for the same color parameter, while different uppercase letters are indicative of significant differences between raw and cooked samples for the same treatment and color parameter (*p* < 0.05).

**Table 5:** Sensory evaluation of pork loin samples during 12 days of refrigerated storage ∗.

Traits (Attributes)	Treatment	Storage time (days)				
		0	3	6	9	12
Color acceptability	C	8.33 ± 0.49 ^c^	7.92 ± 0.29 ^b^	7.58 ± 0.51 ^b^	6.25 ± 0.45 ^a^	5.67 ± 0.49 ^a^
	S	8.08 ± 0.51 ^bc^	7.83 ± 0.39 ^b^	7.42 ± 0.51 ^b^	6.58 ± 0.51 ^a^	5.83 ± 0.39 ^ab^
	SCM	7.25 ± 1.14 ^a^	6.92 ± 1.00 ^a^	6.67 ± 0.78 ^a^	6.42 ± 0.51 ^a^	6.25 ± 0.62 ^bc^
	PM	7.67 ± 0.49 ^ab^	7.50 ± 0.52 ^b^	7.33 ± 0.49 ^b^	7.08 ± 0.51 ^b^	6.42 ± 0.51 ^c^
Tenderness	C	7.25 ± 0.45 ^a^	7.08 ± 0.29 ^a^	6.83 ± 0.39 ^a^	6.42 ± 0.51 ^a^	5.92 ± 0.67 ^a^
	S	7.75 ± 0.45 ^b^	7.58 ± 0.51 ^b^	7.33 ± 0.49 ^b^	6.92 ± 0.51 ^b^	6.58 ± 0.51 ^b^
	SCM	7.67 ± 0.65 ^ab^	7.50 ± 0.52 ^b^	7.25 ± 0.45 ^ab^	6.75 ± 0.45 ^ab^	6.33 ± 0.49 ^ab^
	PM	7.33 ± 0.49 ^ab^	7.25 ± 0.45 ^ab^	7.08 ± 0.29 ^ab^	6.67 ± 0.49 ^ab^	6.25 ± 0.45 ^ab^
Juiciness	C	6.75 ± 0.62 ^a^	6.42 ± 0.51 ^a^	6.17 ± 0.39 ^a^	5.58 ± 0.51 ^a^	4.92 ± 0.29 ^a^
	S	7.33 ± 0.65 ^b^	6.92 ± 0.51 ^b^	6.58 ± 0.51 ^b^	6.17 ± 0.72 ^b^	5.67 ± 0.49 ^b^
	SCM	7.17 ± 0.58 ^ab^	6.83 ± 0.39 ^ab^	6.50 ± 0.52 ^ab^	6.08 ± 0.67 ^ab^	5.42 ± 0.67 ^b^
	PM	6.83 ± 0.72 ^ab^	6.67 ± 0.65 ^ab^	6.42 ± 0.51 ^ab^	5.83 ± 0.58 ^ab^	5.25 ± 0.62 ^ab^
Taste	C	7.75 ± 0.45 ^a^	7.58 ± 0.51 ^a^	7.17 ± 0.39	6.33 ± 0.49 ^a^	5.42 ± 0.51 ^a^
	S	8.50 ± 0.52 ^bc^	8.33 ± 0.49 ^b^	7.83 ± 0.39	6.67 ± 0.65 ^bc^	6.25 ± 0.45 ^ab^
	SCM	8.25 ± 0.45 ^b^	7.92 ± 0.29 ^a^	7.67 ± 0.49	6.83 ± 0.58 ^b^	6.42 ± 0.51 ^b^
	PM	8.67 ± 0.49 ^c^	8.50 ± 0.52 ^b^	8.17 ± 0.39	7.33 ± 0.49 ^c^	6.58 ± 0.51 ^c^
Flavor	C	7.83 ± 0.39 ^a^	7.58 ± 0.51 ^a^	7.17 ± 0.58 ^a^	6.17 ± 0.39 ^a^	4.75 ± 0.45 ^a^
	S	8.33 ± 0.49 ^b^	8.08 ± 0.29 ^b^	7.58 ± 0.51 ^b^	6.67 ± 0.49 ^b^	5.42 ± 0.51 ^b^
	SCM	8.58 ± 0.51 ^b^	8.33 ± 0.49 ^b^	7.92 ± 0.29 ^bc^	7.50 ± 0.52 ^c^	6.75 ± 0.45 ^c^
	PM	8.67 ± 0.49 ^b^	8.42 ± 0.51 ^b^	8.25 ± 0.45 ^c^	7.67 ± 0.49 ^c^	6.92 ± 0.29 ^c^
General acceptability	C	7.58 ± 0.51 ^a^	7.33 ± 0.49 ^a^	6.92 ± 0.67 ^a^	5.92 ± 0.29 ^a^	4.50 ± 0.52 ^a^
	S	8.33 ± 0.49 ^b^	8.17 ± 0.39 ^b^	7.67 ± 0.49 ^b^	6.42 ± 0.51 ^b^	4.83 ± 0.39 ^a^
	SCM	8.08 ± 0.67 ^b^	7.92 ± 0.67 ^b^	7.58 ± 0.51 ^b^	6.83 ± 0.39 ^c^	6.42 ± 0.51 ^b^
	PM	8.42 ± 0.51 ^b^	8.33 ± 0.49 ^b^	7.83 ± 0.39 ^b^	7.25 ± 0.45 ^d^	6.75 ± 0.45 ^b^

∗ C, Control (without marination); S, samples marinated in 3% NaCl solution; SCM, samples marinated in sour cherry juice based marinade; PM, samples marinated in plum juice based marinade; ^a–d^ values with different superscript letters within a column for the same sensory attribute differ significantly (*p* < 0.05).

## Data Availability

No new data were created or analyzed in this study. Data sharing is not applicable to this article.
